# Redox-active conducting polymers modulate *Salmonella* biofilm formation by controlling availability of electron acceptors

**DOI:** 10.1038/s41522-017-0027-0

**Published:** 2017-09-04

**Authors:** Salvador Gomez-Carretero, Ben Libberton, Karl Svennersten, Kristin Persson, Edwin Jager, Magnus Berggren, Mikael Rhen, Agneta Richter-Dahlfors

**Affiliations:** 1 Department of Neuroscience, Swedish Medical Nanoscience Center, Karolinska Institutet, SE-171 77 Stockholm, Sweden; 2 Laboratory of Organic Electronics, Department of Science and Technology, ITN, Linköping University, S-601 74 Norrköping, Sweden; 3 Sensor and Actuator Systems (SAS), Department of Physics, Chemistry and Biology (IFM), Linköping University, 581 83 Linköping, Sweden; 4 Department of Microbiology, Tumor and Cell Biology, Karolinska Institutet, 171 77 Stockholm, Sweden

## Abstract

Biofouling is a major problem caused by bacteria colonizing abiotic surfaces, such as medical devices. Biofilms are formed as the bacterial metabolism adapts to an attached growth state. We studied whether bacterial metabolism, hence biofilm formation, can be modulated in electrochemically active surfaces using the conducting conjugated polymer poly(3,4-ethylenedioxythiophene) (PEDOT). We fabricated composites of PEDOT doped with either heparin, dodecyl benzene sulfonate or chloride, and identified the fabrication parameters so that the electrochemical redox state is the main distinct factor influencing biofilm growth. PEDOT surfaces fitted into a custom-designed culturing device allowed for redox switching in *Salmonella* cultures, leading to oxidized or reduced electrodes. Similarly large biofilm growth was found on the oxidized anodes and on conventional polyester. In contrast, biofilm was significantly decreased (52–58%) on the reduced cathodes. Quantification of electrochromism in unswitched conducting polymer surfaces revealed a bacteria-driven electrochemical reduction of PEDOT. As a result, unswitched PEDOT acquired an analogous electrochemical state to the externally reduced cathode, explaining the similarly decreased biofilm growth on reduced cathodes and unswitched surfaces. Collectively, our findings reveal two opposing effects affecting biofilm formation. While the oxidized PEDOT anode constitutes a renewable electron sink that promotes biofilm growth, reduction of PEDOT by a power source or by bacteria largely suppresses biofilm formation. Modulating bacterial metabolism using the redox state of electroactive surfaces constitutes an unexplored method with applications spanning from antifouling coatings and microbial fuel cells to the study of the role of bacterial respiration during infection.

## Biofilm control: An electrifying effect

Biofilm formation on surfaces can be inhibited or promoted by controlling the availability of electrons and electron-acceptors. This offers a novel way to protect medical devices and machinery from biofilm fouling. It could also assist studies of bacterial metabolism during the formation of biofilms. Agneta Richter-Dahlfors and colleagues at the Swedish Medical Nanoscience Center at Karolinska Institutet investigated the effect with cultured *Salmonella* bacteria. They used electrodes composed of an electrically conducting polymer to maintain the surfaces exposed to bacteria in an electron-rich or electron-deficient state. The research highlights the crucial role of electron transfer in biofilm formation. The rate of biofilm production can be tuned and studied by varying the electron-donating or electron-accepting property of a surface. Healthcare devices, food processing and industrial machinery are potential areas for application of the anti-biofilm effect.

## Introduction

Biofilms represent a form of bacterial growth that enhances survival in wetted natural and artificial environments.^1^ The accompanying resilience, including decreased susceptibility to disinfectants and antibiotics,^1, 2^ increases the need for prevention of biofilm formation, particularly on implanted medical devices and on food contact surfaces. This has prompted a revision of traditional methods and practices, re-evaluating them in relation to bacterial adherence and growth.

Bacteria readily adapt their lifestyles to the local environment. The metabolism of bacteria undergoing planktonic growth differs vastly from bacteria in the biofilm-forming state.^3, 4^ One important determinant is the redox state of the microenvironment in which bacteria exist. Sensing of the redox state in the external environment is used by *Escherichia coli* (*E. coli*) to control the switch between aerobic and anaerobic metabolism using the ArcBA two-component gene regulatory system.^5^ In *Salmonella enterica* serovar *Typhimurium* (*S*. *Typhimurium*) the external redox state plays a major role in bacterial virulence. *S*. *Typhimurium* can survive the oxidative stress inside macrophages by producing an array of enzymes such as superoxide mutases and catalases that inactivate reactive oxygen species.^6^ Periplasmic disulfide isomerases not only protect against redox stress,^7^ but are also required for the production of virulence factors such as Pef fimbrae.^8^ Moreover, disulfide isomerases are required for biofilm formation,^9^ indicating the importance of redox regulation in controlling the bacterial metabolism in this specialized lifestyle.

**Fig. 1:**
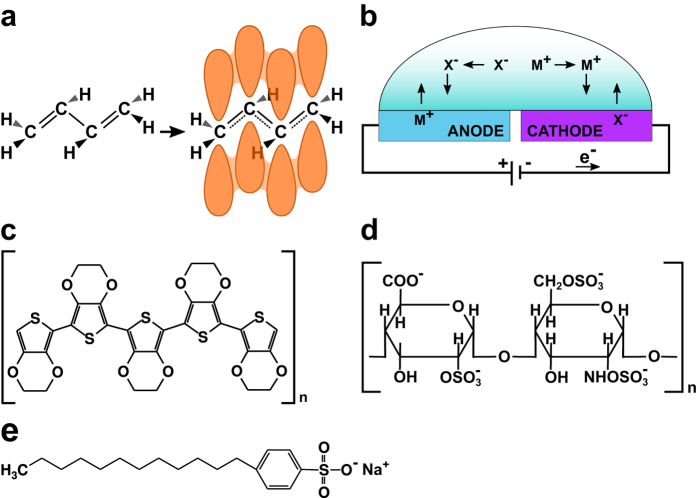
Chemical structures and electrochemical redox reactions in conducting polymers. **a** Chemical structure of the conducting polymer polyacetylene (left) and its resonance hybrid (right) depicting the overlapped p-orbitals of the resultant conjugated system. **b** Flux of ions in a conducting polymer-based electrochemical cell. M^+^ and X^−^ represent positively and negatively charged ions, respectively. The blue color of the anode and purple color of the cathode represent the electrochromism effect occurring in the oxidized and reduced conducting polymer poly(3,4-ethylenedioxythiophene) (PEDOT). **c** Chemical structure of PEDOT. **d** Chemical structure of heparin. **e** Chemical structure of sodium dodecylbenzenesulfonate (NaDBS)

Recent advances in the field of biomaterial research have generated a range of materials whose interaction with biological systems can be finely tuned by changing properties such as surface charge,^10–12^ hydrophobicity,^13^ and roughness.^14^ An interesting group of materials is organic conducting polymers, which can modulate on demand a variety of properties such as hydrophobicity, roughness, redox state and exposed chemical groups.^15–20^ The electrical conductivity of this material arises from their π-conjugated structure, e.g., alternating single and double bonds along the main chain of the polymer (Fig. 1a).^15–17^ The polymer conductivity can be highly increased by chemical or electrochemical oxidation of the polymer chains. This induces positively charged units in the conjugate system, which is equilibrated by negatively charged counter-ions via a process termed doping. A variety of counter-ions can be used to maintain charge neutrality in the conducting polymer, all forming ionic complexes with the polymer chain.

The operation of conducting polymer devices builds on electrochemical redox reactions. When a potential is applied over two conducting polymer electrodes submerged in an electrolyte, ions in the electrolyte migrate towards and into the anode and cathode depending on their charge. These ion fluxes participate in the change of the redox state of the polymer and the maintenance of charge neutrality (Fig. 1b).^21–23^ Anions migrate to the anode to compensate for the oxidation of the polymer, whereas cations migrate to the cathode to compensate for the reduction. Formation of electrical double layers on electrode surfaces is therefore dramatically reduced, which minimizes electrostatic interactions with charged bodies within the electrolyte after electrochemical stabilization of the polymer.^24^ In addition to the electrochemical state of the polymer, its electrical conductivity, its color, and even the exposure of chemical groups, among others, can be tuned in real-time by applying an external, low-volt signal to the conjugated polymer electrode.^15–23, 25^

Thiophene-based polymers, like poly(3,4-ethylenedioxythiophene (PEDOT) (Fig. 1c), are often employed in organic bioelectronic devices. This material has proven biocompatibility, and good stability over a wide pH range.^26, 27^ When epithelial cells were cultivated in a modified cell culture dish coated with doped PEDOT, the redox states of the polymer were shown to dramatically influence the ability of cells to adhere and proliferate. Whereas the reduced, negatively biased electrode surface promoted cell attachment and growth, the oxidized, positively biased electrode surface showed few, often apoptotic, adherent cells.^28, 29^ This resulted from conformational changes in surface-adsorbed proteins induced by the specific redox condition of the oxidized cell-hosting surface.

Redox active proteins and other redox-sensitive compounds are also essential for controlling bacterial metabolism. The presence of such compounds in the extracellular matrix defines the redox potentials in a biofilm and at the interface between a biofilm and electrode surface. These processes have been extensively studied in the microbial fuel cell field, with a specific aim of maximizing the number of electrons transferred to a solid electrode surface in order to enhance electrical power.^30^ However, by focusing solely on power generation, many low efficiency electron transfer processes are overlooked. Studies of bacterial species capable of electrical interactions with surfaces, but generating smaller currents exist but are scarce.^31, 32^ Furthermore, whether modulation of the redox state of a surface can influence the metabolism of bacteria both as individuals and in complex biofilm communities is unknown.

In the present study, we investigate whether the bacterial lifestyle can be tuned by controlling the redox state of the biofilm-supporting surface. By combining disciplines of microbiology, organic bioelectronics, and electrochemistry, we generate redox active surfaces, and we study the effect of the electrochemical state on *S*. *Typhimurium* biofilm formation.

## Results

### Doped PEDOT surfaces for active redox control

**Fig. 2:**
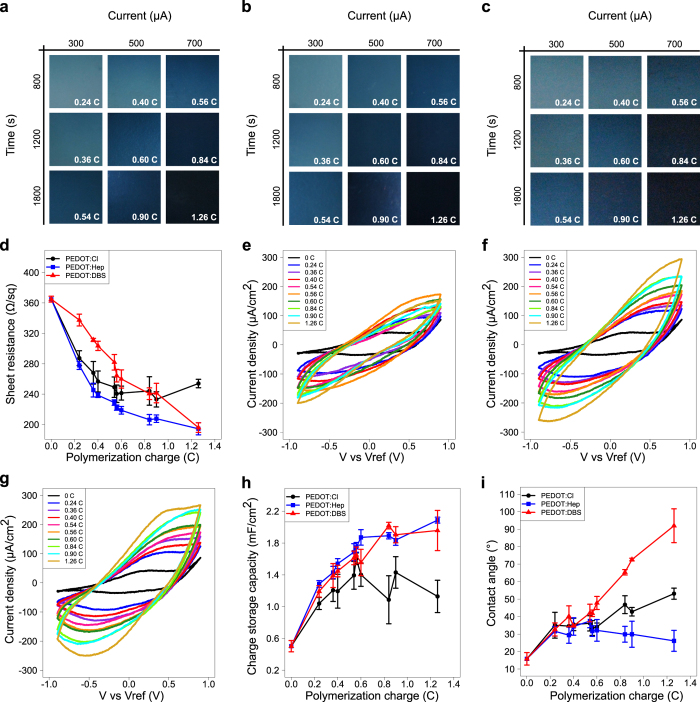
Characterization of electropolymerized composites. **a**–**c** Photographs illustrating the opacity of **a** PEDOT:Cl, **b** PEDOT:Heparin and **c** PEDOT:DBS fabricated for indicated time (s) and currents (µA). The resulting polymerization charge (C) is shown in white on each composite. **d** Electrical sheet resistance (Ω/sq) of PEDOT:Cl, PEDOT:Heparin and PEDOT:DBS composites fabricated at indicated polymerization charges (C). Results expressed as mean ± SEM (*n* = 3). **e**–**g** Cyclic voltammograms of **e** PEDOT:Cl, **f** PEDOT:Heparin and **g** PEDOT:DBS. Each color represents a composite fabricated at specific electropolymerization charges (0.0–1.26 C) as shown in the inset. **h** Charge storage capacity per electrode surface unit (mF/cm^2^), calculated from the cyclic voltammograms shown in (**e**–**g**), for PEDOT:Cl, PEDOT:Heparin and PEDOT:DBS. Results expressed as mean ± SEM (*n* = 3). **i** Surface hydrophobicity, measured as water contact angle (°), of the Orgacon™ starting material (0.0 C) and the PEDOT:Cl, PEDOT:Heparin and PEDOT:DBS composites fabricated at increasing electropolymerization charges. Results expressed as mean ± SEM (*n* = 3)

To create surfaces capable of changing their redox state, we fabricated thin layers of the conducting polymer PEDOT. As dopants, we selected heparin (Fig. 1d) for its common use as catheter coating,^33–35^ and dodecyl benzene sulfonate (DBS) (Fig. 1e) for its common use as antibacterial sanitizer in food industry.^36^ For comparison, Cl^−^ was used as dopant, providing charge equilibration to PEDOT with a minimum of chemical modifications. Films of conducting polymer composites were produced by electrochemical polymerization onto the conducting material Orgacon™, which itself consists of industrially produced PEDOT doped with sodium polystyrene sulfonate (PSS) over a polyethylene terephthalate (PET) substrate. To evaluate the influence of fabrication parameters on the material properties, polymerization was performed using three electrical current intensities (300, 500, and 700 µA) during 800, 1200, and 1800 s time periods. Visual inspection of the nine resulting surfaces of each composite revealed a direct relationship between polymerization charge and opacity, which reflected the thickness of the polymer layer (Fig. 2a–c). The thickest, and therefore darkest surface was generated with a charge of 1.26 Coulomb (C). To obtain quantifiable measures of the integrity of the conjugated backbone of the composites, electrical resistance measurements were performed (Fig. 2d). Starting from a reference value of 364.9 ± 3.9 Ω sq^−1^ (mean ± SEM with *n* = 3), corresponding to the sheet resistance of Orgacon™, a decrease in electrical resistance was observed up to *Q* = 0.6 C for all three composites. This signifies the increase in conductivity as the deposited layer of conducting polymer grows thicker with increasing polymerization charge. Interestingly, while at higher polymerization charges the sheet resistance kept decreasing for PEDOT:Hep and PEDOT:DBS, it remained constant and even slightly increased for PEDOT:Cl. This indicates a certain polymer degradation, possibly by overoxidation during the electropolymerization procedure, of PEDOT:Cl at high polymerization charges.

Luria-Bertani (LB) medium without salt is commonly used as medium for *Salmonella* growth and biofilm formation. We therefore analyzed the electrochemical interactions between this medium and the polymer surfaces. Cyclic voltammetry showed a consistent increase for both the anodic and cathodic currents, indicating progressing charge storage upon polymer oxidation and reduction when an external voltage was applied (Fig. 2e–g). Furthermore, no sharp peaks were observed in the cyclic voltammograms, indicating that the electrical addressing of the electrodes did not degrade the components of the LB medium. This finding was further confirmed with square wave voltammetry on selected surfaces (Fig. S1). Taken together, presented data indicate that PEDOT-based conducting polymer systems are compatible with standard microbiological techniques.

Whereas bacterial adhesion to biotic surfaces often is mediated by fimbriae binding to defined receptors,^37, 38^ initial bacterial interaction with abiotic surfaces is mainly dictated by electrostatic and hydrophobic interactions.^12, 39, 40^ Although electrically neutral, the electrochemical properties of conducting polymers, such as the ability for charge storage, might influence the bacterial behavior at the surface-liquid interface. This in turn could profoundly influence the ability of bacteria to attach. To analyze whether the charge accumulated in the material as a result of the applied voltage was affected by the different doping ions, the charge storage capacity, proportional to the area within the cyclic voltammogram, was calculated for each polymer composite. A similar increase was observed with growing polymerization charges in all three composites up to *Q* = 0.6 C (Fig. 2h). For higher polymerization charges, the increase rate is reduced for PEDOT:Hep and PEDOT:DBS, while a decline is observed in PEDOT:Cl. This is in agreement with data presented in Fig. 2d, confirming the degradation of PEDOT:Cl at large fabrication charges. When polymer degradation does not occur, the electrochemical behavior of the polymer composite appears to be independent of the employed counter-ion for the tested compounds.

The hydrophobic effect, originating from the arrangement of water molecules around the solid-liquid interface, is known to affect bacterial attachment.^39, 40^ To compare the hydrophobicity of the three polymer composites, water contact angles (CA) were measured (Fig. 2i). The control composite PEDOT:Cl showed increased hydrophobicity as polymerization progressed. Starting with a CA of 15.8° ± 3.6° (mean ± SEM with *n* = 3) at 0 C on the Orgacon™ starting material, the CA increased to eventually reach close to 50°. Similar trends were observed for PEDOT:Hep and PEDOT:DBS when charges ≤0.6 C were applied. At higher charges, PEDOT:DBS showed a marked increase in hydrophobicity, with a CA of approximately 90° at *Q* = 1.26 C, possibly due to the increase in surface exposure of the hydrophobic tails of DBS. In contrast, the hydrophilicity of heparin maintained the CA of PEDOT:Hep to around 20°–30° when the higher charges were applied.

When considering all results from the characterization experiments, we conclude that fabrication of polymer composites at 500 µA for 1200 s, corresponding to 0.6 C, allowed for sufficient polymer deposition while maintaining an acceptable transparency in order to facilitate optical microscopy and visual inspection of biofilms. Fabrication at 0.6 C also generated polymer composites with relatively similar charge storage capacity and hydrophobicity, thus giving an opportunity to study the effect of the electrochemical redox state and exposure of chemical functional groups as sole factors affecting biofilm formation. In addition, no polymer degradation was found at *Q* = 0.6 C for any of the three composites. Consequently, this particular set of fabrication parameters were chosen to generate electroactive surfaces for subsequent biofilm studies.

### Bacterial culturing device with integrated redox switching

**Fig. 3:**
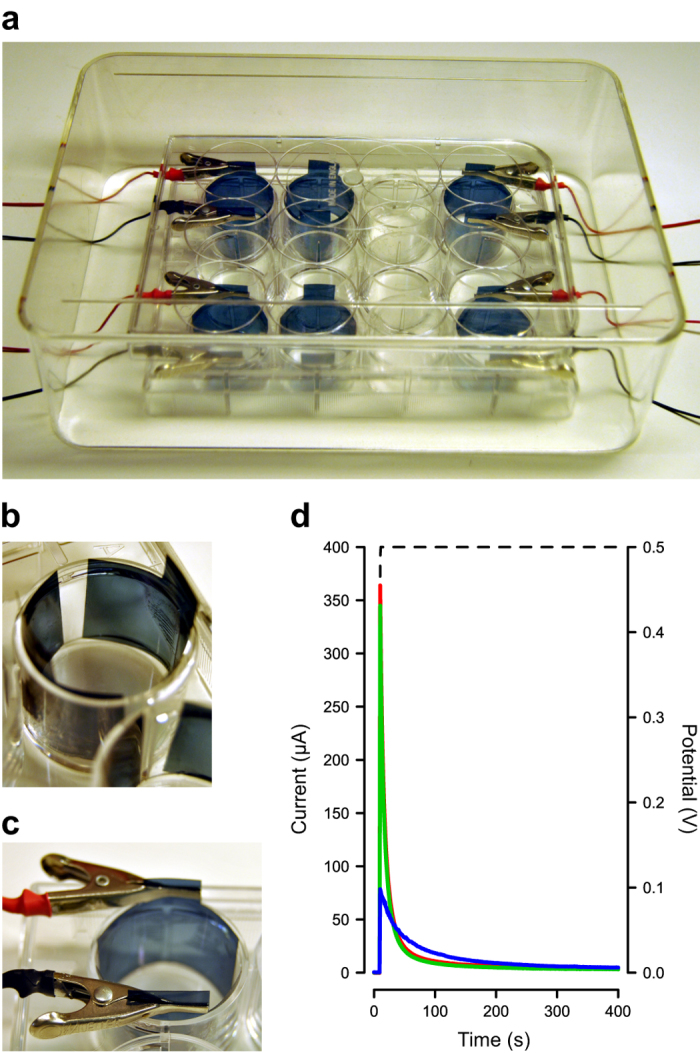
Bacterial cultivation device for integrated redox switching. **a** Photograph of the bacterial cultivation device, which consists of a modified 12-well plate. The four wells in one row are inoculated with the same bacterial culture, and wells in the other row are inoculated with LB without salt. Electrical connections are made to the two outermost wells, enabling redox switching of the wall-attached electrodes. One well is left without electrical connections to provide the unswitched control. The fourth well contains wall-attached, non-conductive polyester surfaces serving as positive control for biofilm formation. The inoculated, electrically addressed device is covered with its lid and an open transparent box placed upside down as extra protection during incubation. **b** Close-up on the positioning of two electrodes of the same PEDOT composite inside a well. **c** Electrical connection of the electrodes made just above the well hinders any contact with the liquid culture. **d** Characterization of the electrical current circulating in a well modified with two electrodes of PEDOT:Cl (blue), PEDOT:Heparin (red) or PEDOT:DBS (green) upon application of a 0.5 V voltage step (dashed line). LB without salt was used as supporting electrolyte

*S*. *Typhimurium* is known to form biofilm on surfaces at the air-liquid interface.^9^ We therefore designed a device with two rectangular surfaces of a PEDOT composite vertically positioned in the wells of a standard 12-well plate (Fig. 3a). To facilitate subsequent analysis, we used polydimethylsiloxane (PDMS) when attaching the flexible surfaces to the walls of the well, thereby allowing their removal after incubation. The shape of the PEDOT composites was designed to ensure that for each well containing two electrodes, the covered area was maximized, minimizing the exposure of uncovered plastic from the walls of the well (Fig. 3b). Electrical connections were made to the PEDOT composites immediately above the well using small planar metal clamps, which did not come into contact with the liquid at any time (Fig. 3c). By applying a low voltage (0.5 V) between the two electrode surfaces when LB medium with or without bacteria was added, we formed an electrochemical cell in the well. The current circulating through the cell was then characterized. An initial, sharp increase was observed in the three composites, which was due to ion fluxes that establish electrical neutrality of the composite, as well as to the rearrangement of charges from the electrical double layers (Fig. 3d, Fig. S2). This was followed by a rapid decay in current, driving towards negligible levels within 1–2 min in all three composites. This decay originated from the capacitive discharge of the electrode double layers together with the completion of the oxidation and reduction processes on the conducting polymers at anode and cathode, respectively.^28^ Particularly, the reduction of the cathode largely decreases its electrical conductivity, increasing the electrical resistance of the complete system.^22, 23, 28^ Collectively, this means that for all composites, it takes at most 2 min for the surfaces to establish their fully reduced or oxidized states following electronic addressing. This very short time frame, compared to 24 h given for bacteria to form biofilms, suggests that bacteria meet a fully reduced or oxidized surface already when initiating the adherence process. As the electrochemical states remain throughout the time given for colonization of the surfaces, our two-electrode set-up represents a defined system permitting the study of biofilm formation on both oxidized and reduced electrodes of one bacterial culture in a single well.

In one row of the device, representing one experiment, three wells were prepared with PEDOT composites: two wells were used for redox switching, one well was left without electrical addressing. The latter, which serves as a control, represents the unswitched, semi-oxidized state of the PEDOT composites, having a majority of oxidized, compared to reduced, polymer chains. One well, containing two rectangular, non-conductive polyester surfaces, was used for comparison of biofilm formed on common consumer plastic material.

### Surface redox states influence biofilm formation

**Fig. 4:**
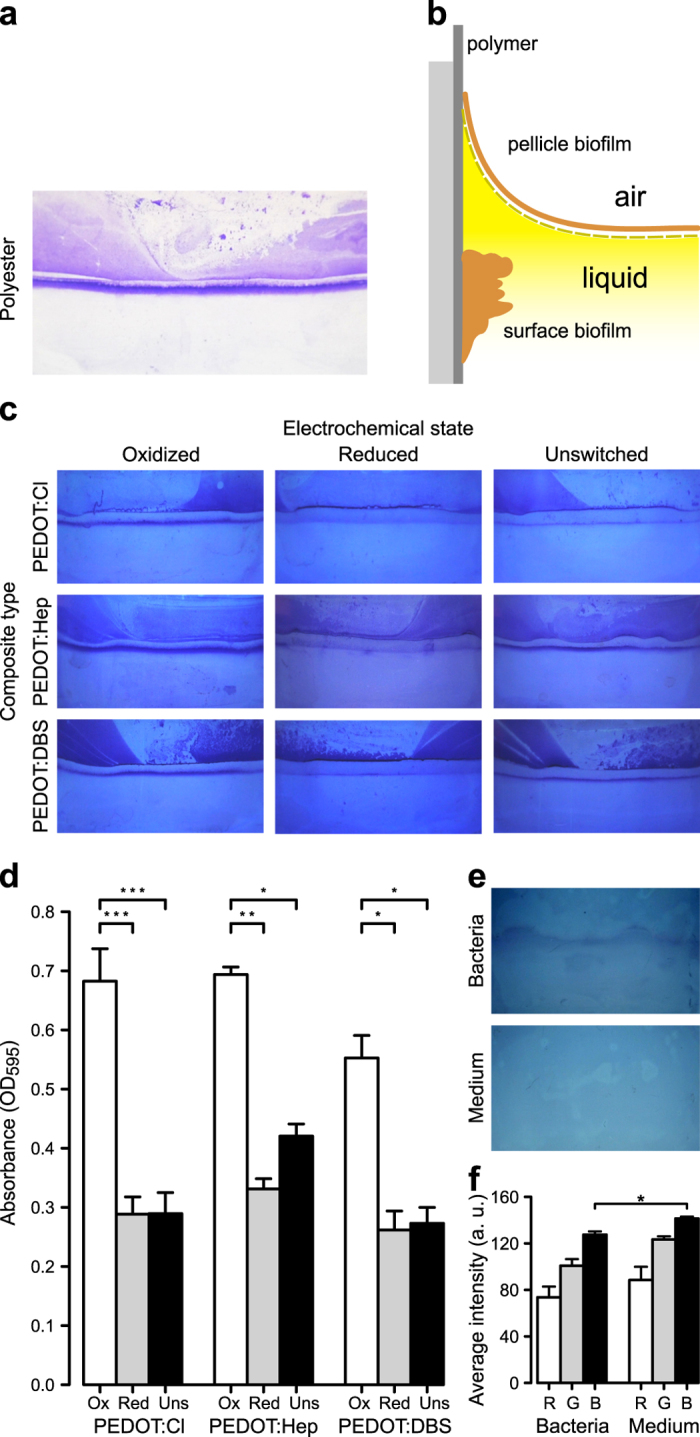
Visualization and quantification of *Salmonella* surface biofilm formed on redox active PEDOT composites. **a** Photograph of a crystal violet-stained polyester surface showing a narrow, distinct purple band (marked with arrow) representing the biofilm formed after 24 h on the surface at the air-liquid interface. Residues from the pellicle biofilm show as smeared, purple stain in the upper half of the surface. **b** Schematic, cross-section representation of surface biofilm and pellicle biofilm at the air-liquid interface in a well from the experimental device. **c** Visual inspection of surface biofilm formed on the three different PEDOT composites in different electrochemical states. Representative photographs of crystal violet-stained PEDOT composites are shown. **d** Quantification of surface biofilms formed on PEDOT composites under different electrochemical conditions. Absorbance at 595 nm was recorded after extraction of crystal violet bound to each surface biofilm. Results are expressed as mean ± SEM. Statistical significance (*p < 0.05, **p < 0.01, ***p < 0.001) from ANOVA analysis and Tukey’s HSD. Ox  =  oxidized composite, Red = reduced composite, Uns = unswitched composite. **e** Photographs of unswitched PEDOT:Cl composites immediately after their removal from a biofilm culture (“Bacteria”) and from LB without salt (“Medium”). **f** Average color intensity of the red (R), green (G) and blue (B) channels of an area at the air-liquid interface in the PEDOT:Cl composites. Results are expressed as mean ± SEM (*n* = 3). Statistical significance (*p < 0.05) from MANOVA analysis and Wilks’ lambda. a.u. = arbitrary unit

To analyze whether the redox states influence the ability of *S*. *Typhimurium* to form biofilm on the polymer surfaces, bacterial cultures were added to the device. Following 24 h of static cultivation, with and without electric addressing as indicated in Fig. 3a, culture supernatants were discarded and each pair of surfaces were removed. Following staining with crystal violet, visual inspection of the conventional polyester control revealed a narrow and discrete purple band with sharp edges, representing the biofilm formed on the surface at the air-liquid interface (Fig. 4a, b).

Macroscopic examination of the electroactive surfaces revealed that different amounts of biomass had formed on polymers with different electrochemical states. On the oxidized PEDOT surfaces, a similarly sized, discrete purple band showed comparable amounts of biofilm on each of the three composites, independent of the doping ions (Fig. 4c). Thinner bands and less biomass were observed on the reduced and the unswitched PEDOT composites, also irrespective of the doping ion.

To perform an unbiased quantification of the biomass, we cut out the strip specifically containing the surface biofilm, extracted the bound crystal violet and performed a spectrophotometric analysis. When comparing data from all surfaces, an obvious pattern emerged (Fig. 4d). The electrochemical state promoting maximal biofilm formation is oxidized PEDOT. Conversely, the reduced surfaces are less supportive of biofilm. Decreases of 57.7% (PEDOT:Cl), 52.3% (PEDOT:Hep), and 52.6% (PEDOT:DBS) in biomass were observed compared to the oxidized states of the respective composite. Unswitched PEDOT composites also showed less biofilm, with decreases in biomass of 57.6% (PEDOT:Cl), 39.4% (PEDOT:Hep), and 50.6% (PEDOT:DBS) compared to the oxidized states. It thus appears that despite differences in electrochemical states of the PEDOT composites, the reduced and unswitched surfaces exert the same effect on bacteria’s biofilm-forming ability. Interestingly, similar experiments performed on indium tin oxide (ITO) electrodes, a commonly used electrically conducting material, showed no significant differences between the anode, the cathode and the unswitched surface (Fig. S3a, b). This shows that electrostatic interactions and galvanotaxis do not significantly contribute to the results obtained with the studied bacterial strain and the employed experimental conditions.

To gain a more comprehensive understanding of the electrochemical interactions that defines the outcome of biofilm formation in our dynamic system, we next analyzed whether metabolically active bacteria influenced the redox state of conducting polymers. As read-out, we utilized the electrochromic properties of PEDOT, meaning that the material undergoes a color change towards dark purple as the polymer becomes electrochemically reduced.^25^ Following 24 h incubations of bacterial cultures in wells containing unswitched PEDOT:Cl, i.e., no externally applied voltage, the color of the material was analyzed and compared to unswitched PEDOT:Cl electrodes from wells containing medium only. Visual inspection showed a dark purple band at the air-liquid interface on electrodes incubated in the presence of bacteria, which contrasted the light blue color observed on electrodes from control wells (Fig. 4e). The purple band matched the position of the biofilm, indicating that polymer reduction was linked to biofilm development and not to planktonic growth. To quantify this color change, we obtained the average intensity of all pixels for the red, green, and blue (RGB) channel in each pixel at the air-liquid interface, using image processing software (Fig. 4f). When comparing the color intensity between electrodes exposed to bacterial cultures and to medium control, a significant decrease was found in the blue channel. The change in the RGB profile of electrodes exposed to bacterial cultures confirmed that metabolically active bacteria within the biofilm were indeed able to reduce the unswitched polymer by transferring electrons to the electroactive material at the location where the biofilm was formed. Our electrochemical cell thus serves as a dual-responsive device, responding to electrical stimuli originating from an external power supply, as well as to the electron transfer from biofilm-forming bacteria. Since the end result in both cases is electrochemical reduction of the polymer, it explains why the externally reduced and unswitched PEDOT provide similarly low support to biofilm formation.

To assess the biofilm-supporting and biofilm–reducing capacity of the electroactive PEDOT surfaces in relation to the biofilm support of conventional polyester, we next analyzed the amount of biofilm formed on rectangular pieces of polyester mounted vertically in wells of the 12-well device. Spectrophotometric analysis of crystal violet extracted from the strip containing the surface biofilm showed an absorbance (OD_595_) of 0.63 ± 0.05 (mean ± SEM). Conventional polyester thus supports biofilm formation to a similarly high degree as oxidized PEDOT surfaces. This leads us to conclude that reduced and unswitched PEDOT present electrochemical states that actively counteract the biofilm forming capacity of *Salmonella*.

## Discussion

Redox activities are known as hallmarks of biofilm formation, with numerous recent studies highlighting the complexity of inter-bacterial redox signaling within the extracellular matrix,^41–43^ as well as the direct or indirect electron transfer to the solid electrodes during bacterial respiration.^44^ This emphasizes the need of enabling technologies for dynamic tuning of the redox events in living biofilms in real-time, in order to promote or inhibit biofilm formation.

**Fig. 5:**
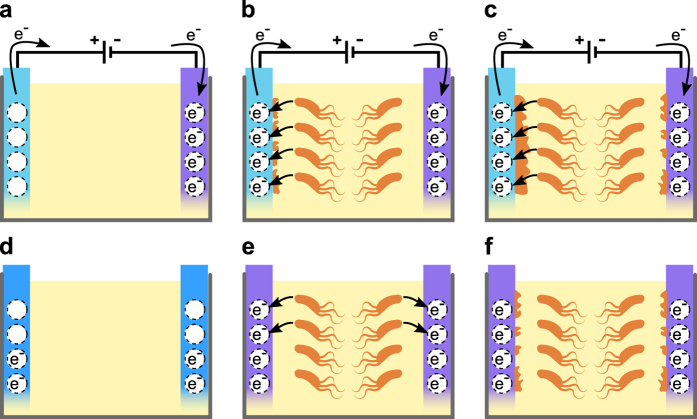
Proposed mechanisms for the electrochemical modulation of biofilm formation. **a** Prior to any bacterial interaction, an external addressing oxidizes the anode (light blue, left electrode), leaving electron holes (dotted-line circle) in the material. Conversely, the cathode (purple, right electrode) becomes reduced, i.e., the material is fully saturated with electrons (dotted-line circle with e^−^). **b** As the external addressing is continuously applied, the anode acts as a continuously renewable electron sink, always providing available sites for bacterial electron transfer (black arrows). This creates a favorable milieu for bacteria to attach and form biofilms on the anode. Conversely, an electron-saturated interface is presented at the cathode, preventing bacterial electron transfer. This hinders bacterial attachment and biofilm formation. **c** The biofilm continues to mature in the anode. In the cathode, alternative factors like hydrophobicity, electrostatic interactions and the use of alternative electron acceptors eventually lead to bacterial attachment and biofilm formation, although at a reduced rate. **d** Pristine, unswitched polymers are in a partially oxidized state (dotted-line circle and dotted-line circle with e^−^) prior to any bacterial interaction. **e** In the absence of an external addressing, bacterial electron transfer quickly reduces the conducting polymer. As the material now presents an electron-saturated interface that prevents bacterial electron transfer, bacterial attachment and biofilm formation are hindered. **f** Similarly to the cathode in **c**, alternative factors like hydrophobicity, electrostatic interactions and the use of alternative electron acceptors eventually lead to bacterial attachment and biofilm formation, although at a reduced rate

We describe the application of conjugated polymers to promote or suppress biofilm-formation by *S*. *Typhimurium*. Using the inherent chromogenicity of the surface, we demonstrate that *S. Typhimurium* can reduce PEDOT in the absence of a directly applied voltage. This underscores the ability of bacteria themselves to influence the surface harboring the biofilm. We saw that when a voltage was applied to PEDOT, large biofilm mass formed on the oxidized electrode compared to the limited amount formed on the reduced. This observation implies that the redox states of electroactive PEDOT surfaces can be tuned to selectively support or inhibit bacterial biofilm formation. Figure 5 shows a schematic representation based on our results, accounting for the electrochemical mechanisms defining the interplay between our engineered biomimetic, electroactive surfaces and bacteria. According to this model, oxidized PEDOT enhances biofilm formation, evidently by functioning as an alternative dynamic electron acceptor (Fig. 5a–c, left electrode). Whether the alternate respiration involves direct electron transfer from bacteria to the material, or indirect via reducing chemical equivalents remains currently unknown.^45, 46^ Since redox reactions in PEDOT are reversible, the material becomes re-oxidized through the removal of accumulated electrons by an external power source. This makes the PEDOT anode a renewable electron sink, continuously available for alternate respiration. By acting as an artificial electron acceptor, biofilm-forming capacity is promoted on oxidized PEDOT.

On the opposite side of the well, bacteria are exposed to a reduced PEDOT electrode (Fig. 5a–c, right electrode). As the external power source maintains PEDOT fully saturated with electrons throughout the time course of experiments, the electrode is prevented from acting as an electron sink. Consequently, bacterial respiration through the electrode is limited, and biofilm formation is hampered. Yet, reduced PEDOT is not inert to bacterial colonization. Residual oxygen and the presence of molecular electron acceptors in the medium may enable limited respiration and finite biofilm formation on the reduced PEDOT.

The natural semi-oxidized state of unswitched PEDOT offers a limited capability of the polymer to act as electron acceptor. At the early stage of colonization, metabolically active bacteria are able to reduce the electrodes, by transferring electrons to the surfaces as the cells undergo respiration (Fig. 5d–f, both electrodes). As no external power source is used, electrons donated by bacteria are not removed, so the reduced condition of the electrodes is maintained during the full length of the experiment. Since this prevents the polymer from acting as an electron sink, respiration is limited and biofilm formation hampered. This explains why similar, low amounts of biomass are formed on unswitched and reduced PEDOT, albeit via different mechanisms: in the former case polymer reduction is caused by bacteria and in the latter by an external power source. This highlights the role of electron transfer in the development of the biofilm.

Interestingly, the above pattern was observed for all composites independent of the nature of doping ions. This verified our initial hypothesis based on material characterization, showing that fabrication of the three composites at 0.6 C enabled isolation of the electrochemical redox state as the sole determinant for bacteria-surface interaction. However, the multifacetted conjugated polymer system also allows for studies on other important material properties in isolation, such as hydrophobicity. The contact angle of PEDOT:DBS alters significantly depending on the polymerization charge applied during fabrication (Fig. 2i). Thus, by controlling the fabrication process, a chemically identical material can be tuned to expose either a hydrophobic or a hydrophilic surface, enabling a study of the influence of hydrophobicity on bacterial-surface interactions.

Our tunable system, in which bacterial metabolism and electroactive surfaces mutually influence each other, opens for relevant applications in basic and applied microbiology research. PEDOT-based surfaces acting as alternative electron acceptors can be used to examine new hypotheses in biofilm research, enabling adjustment of a redox environment to closely mimic the redox potential of biotic surfaces. This allows the development of advanced biomimetic in vitro devices for infection studies. The interactions observed in our system surprisingly mimic the intricate interplay occurring between *Salmonella* and cells of the intestinal mucosa. During infection, *S*. *Typhimurium* causes gut inflammation, leading to generation of reactive oxygen species. This enhances tetrathionate formation, which is used by *Salmonella* as electron acceptor to utilize ethanolamine as carbon source, and thus helps to outcompete local microbiota.^47, 48^ Biofilm formation by *Salmonella* is also sensitive to redox-active compounds in the medium, implying an interplay between redox status and mechanisms responsible for biofilm formation.^9^ By adjusting the redox condition of a defined surface to the redox potential at an infection site, experiments can be performed to study functionality and expression patterns of bacterial virulence factors central to infection pathogenesis and biofilm formation in a relevant yet governable environment.

The availability of suitable electron acceptors is critical to bacterial growth and therefore, the establishment of a biofilm colony. Our demonstrated technology, by which we can tune the availability of electron acceptors by electrically oxidizing or reducing a PEDOT composite, may thus serve important functions in controlling biofouling. To promote biofouling, e.g., in the microbial fuel cell industries, oxidized surfaces can be applied that readily accept electrons from respiring cells facilitating growth and biofilm formation close to the surface. In contrast, antifouling achieved by reduced PEDOT surfaces with a high electron density and a low ability to accept electrons from respiring cells, can be useful to prevent bacterial growth on a variety of objects in the health care sector, as well as in industries such as food processing and paper manufacturing. The list of traditional antifouling methods, exemplified by coatings with tuned surface charge,^10–12^ hydrophobicity,^13^ surface topography^14^ as well as biocidal compounds,^49^ can thus be extended by redox-active conducting polymers communicating with the metabolic systems of bacteria.

## Materials and Methods

### Composite fabrication

Three different PEDOT composites (PEDOT:Cl, PEDOT:Heparin and PEDOT:DBS) were produced by electropolymerization, applying an electrical current for a defined time in specific polymerization solutions. A volume of 500 µl EDOT (3,4-ethylenedioxythiphene, Sigma-Aldrich, Stockholm, Sweden) was added to 50 ml solutions of 0.1 m NaCl (Sigma-Aldrich), 5 mg ml^−1^ porcine NaHeparin (Sigma-Aldrich) or 0.1 m NaDBS (sodium dodecylbenzenesulfonate, Sigma-Aldrich) in MiliQ water (Merck, Solna, Sweden), and solutions were stirred overnight to ensure sufficient EDOT dilution before use. Electropolymerization was performed with a potentiostat (Reference 600, Gamry Instruments, Warminster, Pennsylvania, USA) in a three electrodes configuration, with a platinum grid as counter electrode and a Ag/AgCl electrode as reference. The PEDOT composites were deposited over working electrodes consisting of (width x length) 3.5 × 4.5 cm (13.3 cm^2^ of immersed area) pieces of Orgacon™ EL-350 (PEDOT:PSS-coated PET, Agfa Gevaert, Mortsel, Belgium). Before use, the Orgacon™ working electrodes were briefly washed in acetone (Sigma-Aldrich), thoroughly rinsed with de-ionized water and dried with compressed air. A similar procedure was applied to the resulting composites.

### Sheet resistance

From each PEDOT composite, surfaces sized 2.5 × 2.5 cm were excised, and electrical resistance was measured at a minimum of five different locations per surface using a four points probe (Guardian SRM-232, Guardian Manufacturing, Cocoa, Florida, USA) connected to a source-meter (Keithley 2602A, Tektronix, Beaverton, Oregon, USA). Results expressed as mean ± SEM with *n* = 3. This sample size provided a significance level ≤ 0.05 and statistical power ≥ 0.8.

### Cyclic voltammetry

A potentiostat (Reference 600, Gamry Instruments, Warminster, Pennsylvania, USA) with a three electrodes set-up was employed, with a platinum grid as counter, a Ag/AgCl electrode as reference and 2.5 × 2.5 cm pieces (4.5 cm^2^ of immersed area) of the PEDOT composites to be analyzed as working electrodes. The electrochemical response of each composite was analyzed from −0.9 to 0.9 V using a scan rate of 50 mV s^−1^. LB without salt was used as supporting electrolyte to study the composite behavior in the bacterial growth medium. The charge storage capacity was calculated using formula (1).

1 $$C = \frac{1}{{2v({V_{{{\max}}}} - {V_{{{\min}}}})}}{\oint} {J(V)dV}$$

*C* is the charge storage capacity (mF cm^−1^), *J* is the current density (mA cm^−1^), *v* is the scan rate (V s^−1^) and *V*_max_ and *V*_min_ are the voltage limits (V). Results expressed as mean ± SEM with *n* = 3. This sample size provided a significance level ≤0.05 and statistical power ≥0.8.

### Contact angle

A goniometer (ramé-hart 190, ramé-hart, Succasunna, New Jersey, USA) was used to measure the contact angle of 5 µl MiliQ water droplets deposited on the surfaces. Calculations were performed with the accompanying software. The contact angle was characterized with one droplet per surface. Results expressed as mean ± SEM with *n* = 3. This sample size provided a significance level ≤0.05 and statistical power ≥0.8.

### Assembly of the biofilm device for integrated redox switching

Two surfaces of the same material (PEDOT:Cl, PEDOT:Heparin, PEDOT:DBS or polyester) were cut (3 × 2.4 cm) with a centrally located tab (1 × 0.5 cm) defining the upper end of the surface. The pair of surfaces was glued opposite to each other in a well of a 12-well plate (COSTAR 3513, Corning Incorporated, Corning, New York, USA) using PDMS (prepared from SYLGARD^®^ 184 silicone elastomer kit, Dow Corning, Midland, Michigan, USA) to allow later removal after biofilm had formed. For each experiment two rows of four modified wells were produced. For each row, three wells were prepared with two electrodes of the same PEDOT composite. For the remaining well, two polyester (Rosinco AB, Filipstad, Sweden) films shaped, washed, rinsed and dried similarly to the conducting composites were employed as non-conductive references. Biofilm devices were stored overnight at 70 °C before use.

### Characterization of the circulating current in a modified well

A volume of 3.5 ml of Luria-Bertani (LB) without salt was added as supporting electrolyte to the wells of the assembled biofilm device. A custom made LabVIEW program was used to control a source-meter (Keithley 2602A, Tektronix, Beaverton, Oregon, USA) for the application and recording of the electrical signals. After 10 s of equilibration time, a voltage of 0.5 V was applied for approximately 2 h, recording both the applied voltage and the resultant current at every second. Only the first 400 s are shown, after which the responses remained essentially constant.

### Biofilm culture

We used *S. enterica* serovar *Typhimurium* strain 14028 (ATCC, Manassas, Virginia, USA), a smooth *Salmonella* strain with neutral surface charge, in order to minimize any influence of charge-dependent movement in an electric field.^50, 51^ Bacteria were cultured in LB medium or in LB without salt as indicated. A 14028 overnight culture (shaking, 37 °C) in LB was diluted 1:100 in LB without salt and incubated (shaking, 37 °C) to reach OD_600_ = 0.7. Following 1:10 dilution in LB without salt, the culture was used in the biofilm experiments. The experiment was started by electrically addressing with 0.5 V (Hewlett Packard E3632A power source, Hewlett-Packard, Palo Alto, California, USA) two of the three wells modified with PEDOT composites in both rows of the biofilm device using small clamps connected to the tabs on the upper end of the composites. Immediately thereafter, 3.5 ml of bacterial culture was transferred to each of the four wells in one row of the modified 12-well plate, whereas 3.5 ml LB without salt (no bacteria) was added to each well in the other row to control for sterility and background staining. The 12-well plate was covered with its lid and, for additional protection, with an open transparent box placed upside-down. The plate was then left to culture for 24 h at 28 °C under static conditions. The two-electrode conducting polymer electrochemical cell was preferred over a traditional three-electrode setup with a reference electrode and a non-reactive auxiliary electrode, as it allowed us to study the relative differences between bacterial colonization at the oxidized and reduced electrodes in the same bacterial culture. This minimizes the risk of different conditions in separate cultures, thus focusing our experiments to the study of the physico-chemical interactions between bacteria and the polymer. The two-electrode setup is also best suited to ensure sterile conditions during the 24 h incubation. Moreover, the absence of metal-based reference and pseudo-reference electrodes prevented any possible uncontrolled release of metal ions into the bacterial culture. The electrochromic response of the composites was used to qualitatively confirm the correct voltage bias in the system.

### Crystal violet biofilm analysis

After culturing, the medium was decanted and wells were gently rinsed with phosphate-buffered saline (PBS, pH 7.4) (Medicago AB, Uppsala, Sweden). The surfaces were detached from the wells and immersed in a 0.1% (w/v) solution of crystal violet (Sigma-Aldrich) stain in MiliQ water. After 10 min at room temperature, surfaces were carefully rinsed with PBS, left to dry for 2 h, and then photographed using a backlight for qualitative assessment of the formed biofilms. To maximize the sensitivity of quantification, we manually cut out a 0.3 cm-wide strip containing the surface biofilms formed at the air-liquid interface. The same procedure was applied to the background staining control surfaces. Strips were immersed in 400 µl of 95% ethanol (Kemetyl AB, Jordbro, Sweden) in closed tubes for 1 h. The extracted crystal violet was analyzed in duplicates measuring the optical absorption at 595 nm in a microplate reader (Synergy Mx, BioTek, Winooski, Vermont, USA). Presented results correspond to the difference of absorbance between the biofilm samples and corresponding controls that accounted for the background staining of the surfaces. Results are expressed as mean ± SEM, with *n* = 3 for PEDOT:Cl, PEDOT:Heparin and PEDOT:DBS and *n* = 9 for polyester. This sample size provided a significance level ≤ 0.05 and statistical power ≥0.8. As a polyester positive control was included in every prepared plate, the sample size for polyester was *n* = 9. Statistical analysis were performed using ANOVA analysis and Tukey’s HSD as data is considered to fulfill the assumptions required by this test. Similar variances were obtained within each group.

### Electrochromism analysis

A volume of 3.5 ml of culture of strain 14028 or of medium only were added to wells modified with PEDOT:Cl. This was incubated for 24 h similar as above, however, without electrical addressing. Then, wells were gently washed with PBS before surfaces were removed and immediately photographed using a backlight. Notice that no staining was applied. The average intensity of the red, green and blue channels in the area at the air-liquid interface of each specimen were obtained with ImageJ (http://imagej.nih.gov/ij/). Results are expressed as mean ± SEM, with *n* = 3. This sample size provided a significance level ≤0.05 and statistical power ≥0.8. Statistical analysis was performed using MANOVA analysis and Wilks’ lambda, as data is considered to fulfill the assumptions required by this test. Similar variances were obtained within each group.

### Data availability

The authors declare that data supporting the findings of this study are available within the paper and its supplementary information.

## Electronic supplementary material

Supplementary Information

## Electronic supplementary material

**Supplementary Information** accompanies the paper on the *npj Biofilms and Microbiomes* website (doi: 10.1038/s41522-017-0027-0).
