# Author Correction: Redox-active conducting polymers modulate *Salmonella* biofilm formation by controlling availability of electron acceptors

**DOI:** 10.1038/s41522-018-0061-6

**Published:** 2018-08-07

**Authors:** Salvador Gomez-Carretero, Ben Libberton, Karl Svennersten, Kristin Persson, Edwin Jager, Magnus Berggren, Mikael Rhen, Agneta Richter-Dahlfors

**Affiliations:** 1 Department of Neuroscience, Swedish Medical Nanoscience Center, Karolinska Institutet, SE-171 77 Stockholm, Sweden; 2 Department of Science and Technology, Laboratory of Organic Electronics, ITN, Linköping University, S-601 74 Norrköping, Sweden; 3 Department of Physics, Chemistry and Biology (IFM), Sensor and Actuator Systems (SAS), Linköping University, 581 83 Linköping, Sweden; 4 Department of Microbiology, Tumor and Cell Biology, Karolinska Institutet, 171 77 Stockholm, Sweden

**Correction to:** *npj Biofilms and Microbiomes*; 10.1038/s41522-017-0027-0; Published online 4 September 2017

In the original published article, the author list did not include Karl Svennersten, Kristin Persson, Edwin Jager and Magnus Berggren. After publication, we were notified by the corresponding author that the author list did not accurately reflect the contributions made, and these authors have been added to the author list. The original “Author Contributions” stated that “S.G.C., M.R., and A.R.D. designed research; S.G.C. performed all experiments…” this has been updated to read “S.G.C., K.S., K.M.P., E.W.H.J., M.B., M.R., and A.R.D. designed research; K.S., K.M.P., and E.W.H.J. performed experiments; S.G.C. performed all reported experiments…”. The “Acknowledgements” previously read “We thank K. Svennersten, A. Kader, K. Persson, and M. Berggren for fruitful discussions, and S. Löffler for insightful comments on the manuscript…” and have been updated to state “We thank A. Kader for fruitful discussions and S. Loffler for insightful comments on the manuscript…”. The “Competing Interests” section did not require any amendments. All authors have agreed with this correction statement and authorship change.

