# Supplementary material for: Redox-active conducting polymers modulate Salmonella biofilm formation by controlling availability of electron acceptors
Source: NPJ Biofilms Microbiomes. 2017 Sep 4;3:19. doi: 10.1038/s41522-017-0027-0 (PMC5583241; doi:10.1038/s41522-017-0027-0)
Supplement: Supplementary file 1 — Supplementary Information [file 41522_2017_27_MOESM1_ESM.docx]

**SUPPLEMENTARY INFORMATION**

**Redox-active conducting polymers modulate Salmonella biofilm formation by controlling availability of electron acceptors**

Salvador Gomez-Carretero^1^, Ben Libberton^1^, Mikael Rhen,^1, 2^

Agneta Richter-Dahlfors*^,1^

^1^ Swedish Medical Nanoscience Center, Department of Neuroscience, Karolinska Institutet, SE-171 77, Stockholm, Sweden

^2^ Department of Microbiology, Tumor and Cell biology, Karolinska Institutet, SE-171 77, Stockholm, Sweden

* Corresponding author Agneta Richter-Dahlfors

Karolinska Institutet,

Swedish Medical Nanoscience Center

Department of Neuroscience

Retzius väg 8, SE- 17177

Stockholm, Sweden

Tel: +46 8 5248 7425

email: [Agneta.richter.dahlfors@ki.se](mailto:Agneta.richter.dahlfors@ki.se)

**Supplementary Figure 1**

**Square wave voltammetry as complement to cyclic voltammograms**

To characterize the response of the cyclic voltammograms (Fig. 2e-g) in further detail, square wave voltammetry was performed on PEDOT:Cl, PEDOT:Hep and PEDOT:DBS fabricated at Q = 0.6 (1200 s, 500 µA). A potentiostat (Reference 600, Gamry Instruments, Warminster, Pennsylvania, USA) with a three-electrodes setup was employed, with a platinum grid as counter, a Ag/AgCl electrode as reference and 2.5 cm x 2.5 cm pieces (4.5 cm^2^ immersed area) of the PEDOT composites to be analyzed as working electrodes. The electrochemical response of each composite was analyzed from - 0.9 V to 0.9 V using an amplitude of 25 mV, a frequency of 25 Hz and a pulse step of 2 mV. LB without salt was used as supporting electrolyte.

Square wave voltammetry largely reduced the background current in respect to the cyclic voltammograms, as noted by the different range of the ordinate in the two methods (Fig. S1 compared to Fig. 2e-g). This shows that square wave voltammetry has an increased sensitivity to electrochemical reactions in the medium, as compared to the measurements performed by cyclic voltammetry. An increasing line was observed for the three polymer composites due to the different dynamics of the anodic and cathodic currents. Importantly, no peaks were observed. As this confirms the absence of sharp peaks in the cyclic voltammograms, these results show that the electrical addressing of the conducting surfaces did not degrade the components of the LB medium.


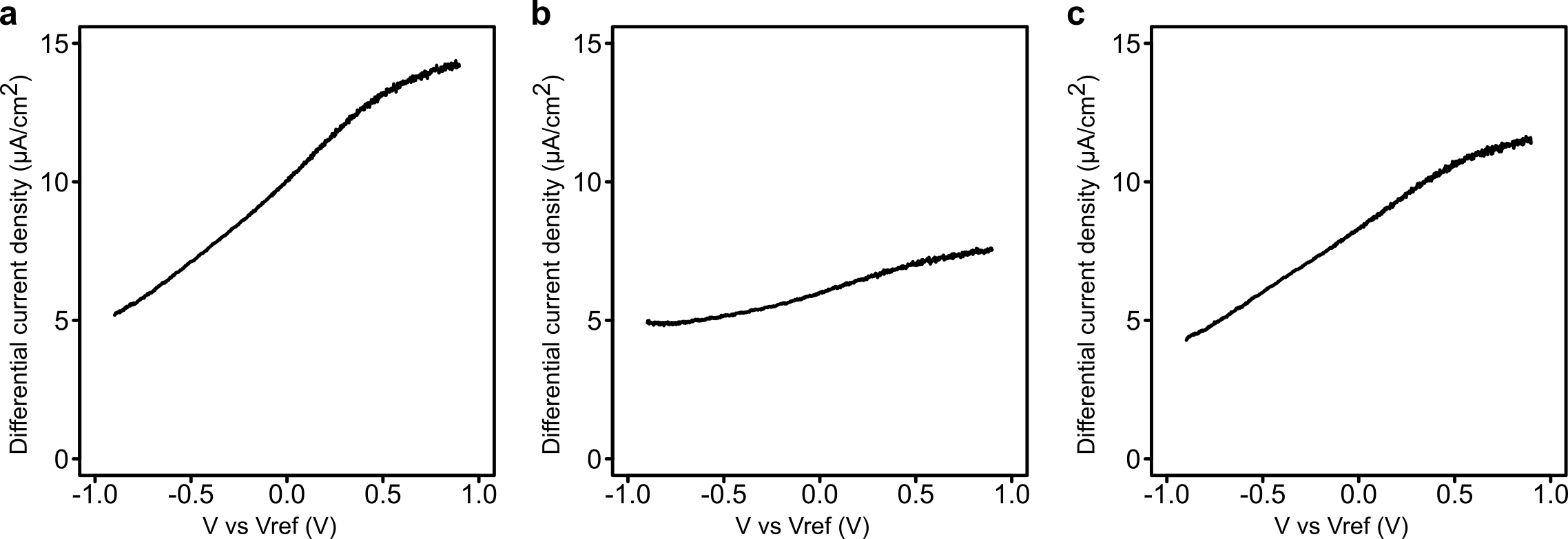


**Supplementary Figure 1. Electrochemical interactions between the conducting polymer composites and LB medium without salt.** (**a**-**c**) Square wave voltammetry analysis of (**a**) PEDOT:Cl (**b**) PEDOT:Heparin and (**c**) PEDOT:DBS surfaces synthetized with a charge of 0.6 C.

**Supplementary Figure 2**

**Characterization of the circulating current in a modified well**

To analyze whether the presence of bacteria influenced the initial electrical response of the system, an experiment similar to the one reported in Fig. 3d was performed, but now using LB medium inoculated with *Salmonella*. A custom made LabVIEW program was used to control a source-meter (Keithley 2602A, Tektronix, Beaverton, Oregon, USA) for the application and recording of the electrical signals. After 10 s of equilibration time, a voltage of 0.5 V was applied for approximately 2 h. The applied voltage and the resultant current were recorded at every second.

Figure S2 shows the first 400 s, since the responses were essentially constant for the remaining part of the 2 h experiment. The curves showed an initial current increase, due to ion fluxes establishing electrical neutrality of the composite, as well as to the rearrangement of charges from the electrical double layers. This was followed by a rapid current decay. This pattern mimicked the pattern seen in the electrical current recordings of electrodes in LB medium without bacteria (Fig. 3d).


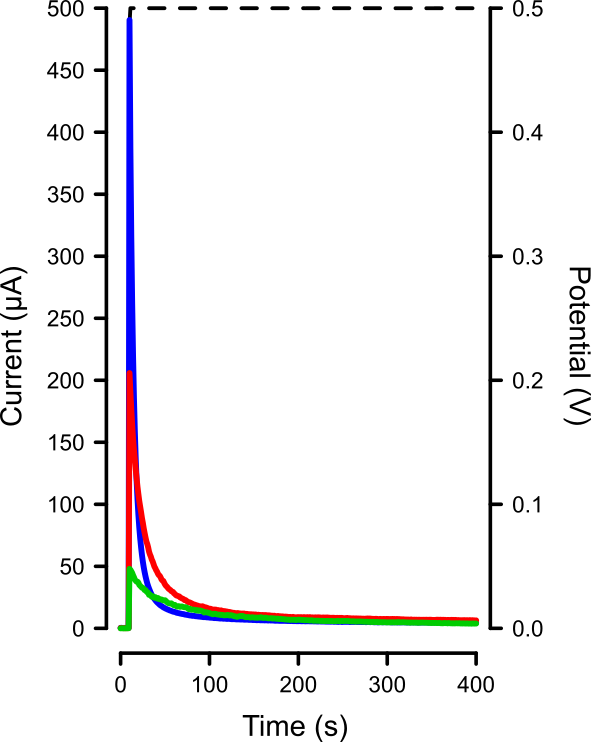


**Supplementary Figure 2. Characterization of the electrical current circulating in wells containing LB medium inoculated with *Salmonella*.** Each well contains two electrodes of PEDOT:Cl (blue), PEDOT:Heparin (red) or PEDOT:DBS (green), which are addressed with a 0.5 V voltage step (dashed line).

**Supplementary Figure 3**

**Characterization of bacterial attachment on ITO surfaces**

As comparison to the bacterial behavior on an electrically conducting material different from conducting polymers, bacterial attachment was tested on ITO (indium tin oxide). The custom-designed culturing device was prepared using ITO (Sigma-Aldrich, Stockholm, Sweden) as electrode material as replacement for the PEDOT composite, including an unswitched ITO control. All other experimental procedures were performed as described in Materials and Methods. The applied voltage was 0.5 V in addressed biofilm cultures.

Visual inspection of the ITO electrodes after staining with crystal violet revealed the formed surface biofilm as a narrow and discrete purple band with sharp edges (Fig. S3a). No significant differences were found between the anode, the cathode and the unswitched surface (Fig. S3b). This result shows that bacterial cells are not significantly driven towards a positively biased electrode by phenomena such as electrostatic attraction or bacterial galvanotaxis.


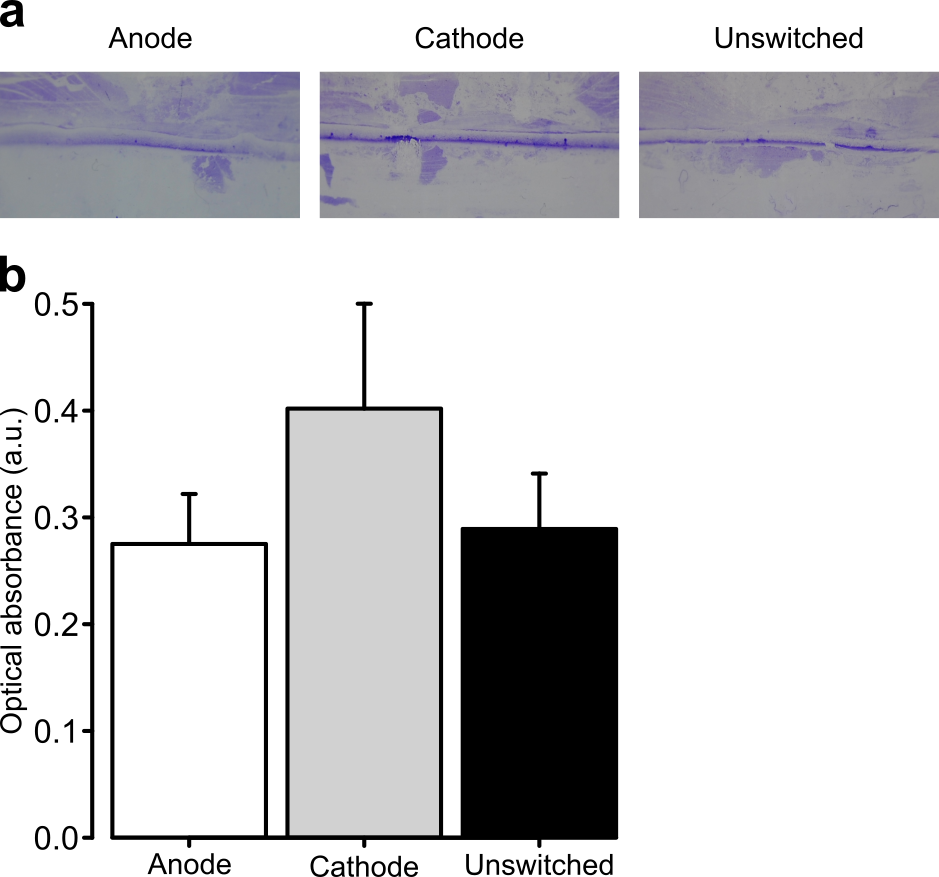


**Supplementary Figure 3. Visualization and quantification of *Salmonella* biofilm formed on ITO surfaces.** (**a**) Visual inspection of crystal violet-stained surface biofilm formed on the ITO anode, cathode and the unswitched ITO control. Representative photographs of ITO surfaces under the different conditions are shown. (**b**) Quantification of surface biofilms formed on the ITO anode, cathode and the unswitched ITO control. Absorbance at 595 nm was recorded after extraction of crystal violet bound to each surface biofilm. Results are expressed as mean ± SEM (n=3).
